# Elevated Transforming Growth Factor-*β*2 in the Aqueous Humor: A Possible Explanation for High Rate of Capsular Contraction Syndrome in High Myopia

**DOI:** 10.1155/2016/5438676

**Published:** 2016-01-28

**Authors:** Keke Zhang, Xiangjia Zhu, Minjie Chen, Xinghuai Sun, Jin Yang, Peng Zhou, Yi Lu

**Affiliations:** ^1^Department of Ophthalmology, Eye and Ear, Nose, and Throat Hospital, Fudan University, 83 Fenyang Road, Shanghai 200031, China; ^2^Key Laboratory of Myopia, Ministry of Health, 83 Fenyang Road, Shanghai 200031, China; ^3^Key Laboratory of Visual Impairment and Restoration of Shanghai, Fudan University, 83 Fenyang Road, Shanghai 200031, China; ^4^Department of Ophthalmology, Parkway Health, Specialty and Inpatient Center (Luwan), 170 DanShui Road, Floor 3, Shanghai 200020, China; ^5^Parkway Health Hong Qiao Medical Center, 2258 Hong Qiao Road, Shanghai 200335, China

## Abstract

*Purpose.* The purpose of the study was to elucidate the role of transforming growth factor-beta 2 (TGF-*β*2) in the development of high myopic capsular contraction syndrome.* Methods.* Nineteen cases of capsular contraction syndrome, including 14 with high myopia, were collected, and their clinical data were reviewed. Aqueous humor and anterior capsular membranes were obtained during capsulotomy. Hematoxylin-eosin and immunohistochemical staining with anti-TGF-*β*2 antibody were performed on capsular membranes. TGF-*β*2 levels in aqueous humor were assayed using ELISA and western blot.* Results.* High myopia was significantly associated with the incidence of capsular contraction syndrome (odds ratio: 14.74, *P* < 0.001, 95% CI: 5.29–41.05). Histopathological analysis revealed proliferation of fibroblast-like lens epithelial cells on the shrunken anterior capsule, labeled with TGF-*β*2 antibodies. ELISA and Western blot showed higher levels of TGF-*β*2 in aqueous humor of patients with capsular contraction syndrome and high myopia.* Conclusions.* High myopia is a risk factor for capsular contraction syndrome. Elevated TGF-*β*2 levels in high myopic cataract patients may play an important role in the pathogenesis of capsular contraction syndrome.

## 1. Introduction

Capsular contraction syndrome (CCS) is characterized by shrinkage and whitening of the anterior capsular opening [1]. It is an uncommon complication after cataract surgery. CCS patients may have impaired visual acuity [2] secondary to opacity of the anterior capsular membrane in the pupillary area and intraocular lens (IOL) decentration within the capsular bag [3].

Previous studies [4] have demonstrated that CCS develops from shrinkage of the capsulorhexis, induced by the proliferation of human lens epithelial cells (LECs) and formation of myofibroblasts. Transforming growth factor-*β*2 (TGF-*β*2) is the key cytokine mediating epithelial-myofibroblast transdifferentiation [5].* In vitro* experiments have shown that addition of TGF-*β*2 to the FHL124 cell line [6] and cultured rat lenses [7] resulted in the formation of myofibroblasts. TGF-*β*2 may also play a role in posterior capsule opacification (PCO) [8] and anterior subscapular cataract (ASC) [9]. Therefore, TGF-*β*2 expression in aqueous humor may increase the risk of CCS.

CCS is also reportedly more common in cataract patients with high myopia [10], retinitis pigmentosa [11], diabetes, and pseudoexfoliation syndrome [1, 11]. High myopia, defined as myopia of −6.0 diopters or more [12], is characterized by axial elongation of the eyeball.

As high myopia is more common in Asian populations than in other ethnic groups, CCS cases in Asia are probably different from the Western cases. As reported in previous studies, elevated expression of TGF-*β*2 was found in the posterior remodeling area of sclera in high myopic eyes [13]. Altered TGF-*β*2 in the anterior chamber of high myopic eyes may affect the pathogenesis of CCS. Therefore, we investigated the clinical features of 19 cases of CCS and analyzed the aqueous humor TGF-*β*2 concentrations and histopathological changes in the anterior capsular membranes, to determine the factors contributing to the high rate of CCS in high myopic cataract (HMC) patients.

## 2. Materials and Methods

### 2.1. Patient and Sample Collection

All tissues obtained during surgery were handled in accordance with the tenets of the* Declaration of Helsinki*. The Ethics Committee of the Eye and ENT Hospital, Fudan University, Shanghai, China, approved our collection and use of aqueous humor and anterior capsular membranes from patients undergoing cataract surgery. Informed consent was obtained from all participants.

Based on previous studies [2], patients with a contracted capsulorhexis opening area of <10 mm^2^ were diagnosed with CCS. Patients with an axial length of ⩾26 mm were diagnosed with high myopia. In this study, 19 eyes of 19 CCS cases, including 14 with a medical history of high myopia, were recruited between September 1, 2012, and March 31, 2013. Follow-up tables were used to retrieve patients' clinical data and to calculate the rate of CCS. Before surgery, a thorough ophthalmic examination was performed. The preoperative exclusion criteria were a history of previous ocular surgery; glaucoma; uveitis; or systemic diseases, such as diabetes mellitus.

Aqueous humor samples were obtained from CCS patients before the operation of capsulotomy in the second surgery (not in the primary cataract surgery). Immediately after the incision was made, the aqueous humor samples (50–150 *μ*L) were aspirated using a 1 mL syringe with a 30-gauge needle, aliquoted into sterilized ethylenediaminetetraacetic acid- (EDTA-) coated microtubes and centrifuged at 400 ×g for 5 min. Then, the tissue samples of shrinked anterior capsular membrane after capsulorhexis in the primary cataract surgery were also collected during the capsulotomy in the second surgery (not in the primary cataract surgery). The cell-free supernatant and anterior capsular membranes were immediately stored at −80°C until use.

### 2.2. Histopathological Analysis of Anterior Capsular Membranes from the CCS Group

Histopathological analysis, including hematoxylin-eosin (HE), periodic acid-Schiff (PAS), and immunohistochemical (IHC) staining, was performed on the anterior capsular membranes from CCS cases. Capsular membranes were washed in sterile balanced saline to eliminate blood and viscoelasticity, fixed in 10% neutral-buffered formalin (Sigma-Aldrich Corporation, St. Louis, MO, USA) for 24 h, dehydrated, and embedded in paraffin wax. Serial transverse sections (6 *μ*m thick) were prepared on a microtome (Leica Mikrosysteme Vertrieb GmbH, Wetzlar, Germany), mounted on positively charged glass slides, and stained with HE or PAS or used for IHC analysis. For the IHC analysis, rehydrated slices were heated in 10 *μ*M citrate buffer (pH 6.0) for antigen retrieval, incubated with 3% hydrogen peroxide, and blocked with normal goat serum. The sections were incubated with the mouse monoclonal anti-TGF-*β*2 (dilution factor: 1 : 200; ab36495; Abcam, Cambridge, UK) primary antibody at 4°C overnight. Next, the sections were incubated at 37°C for 1 h with a goat anti-mouse immunoglobulin G- (IgG-) HRP (dilution factor: 1 : 200; ab136815; Abcam) secondary antibody. Positive signals in the sections were visualized after incubation with 3,3-diaminobenzidine (DAB) solution as the substrate. Sections reacting with DAB, in which the primary antibody was omitted, were used as the negative controls. All sections were observed with a microscope (DMI3000B; Leica Mikrosysteme Vertrieb GmbH) by an experienced pathologist who was blinded to patients' diagnosis.

### 2.3. Enzyme-Linked Immunosorbent Assay (ELISA) of TGF-*β*2 Concentrations in the Aqueous Humor

TGF-*β*2 levels in the aqueous humor samples of each group were determined using a commercially available ELISA kit (DY302; R&D Systems Inc., Minneapolis, MN, USA). A 96-well plate was coated with diluted human TGF-*β*2 monoclonal antibody, followed by addition of aqueous humor samples and incubated for 2 h at room temperature. Next, each well was washed and incubated with a working dilution of streptavidin-horseradish peroxidase (HRP) substrate solution, followed by stop solution, based on the manufacturer's instructions. In all assays, the mean optical density at 450 nm in duplicate wells containing only the diluent as the negative control was subtracted from the test readings. The TGF-*β*2 concentrations were calculated from standard curves.

### 2.4. Western Blot Analysis of TGF-*β*2

After determining the protein concentration by Quick Start Bradford Protein Assay Kit (500-0202, Bio-Rad, Hercules, CA, USA), each vial of selected aqueous humor sample was supplemented with 5x SDS-PAGE loading buffer and denatured under 100°C for 5 min. The samples were separated by 15% gradient acrylamide sodium dodecyl sulphate-polyacrylamide gel electrophoresis gel. Protein bands were transferred onto a PVDF blotting membrane (Millipore, Bedford, MA, USA) and subjected to immunolabeling using primary mouse anti-human antibodies for TGF-*β*2 (1 : 1000 dilution, ab36495, Abcam, Cambridge, MA, USA) and GAPDH (1 : 1000 dilution, ab8245, Abcam, Cambridge, MA, USA). The membranes were incubated with rabbit anti-mouse IgG-horseradish-peroxidase- (HRP-) conjugated secondary antibody (1 : 1000 dilution, ab6721, Abcam, Cambridge, MA, USA) for 30 min at room temperature. Immunoblotted bands were revealed by enhanced chemiluminescence reagent (Pierce ECL Western Blotting Substrate, Thermo Fisher Scientific, Waltham, MA, USA). The intensity of bands was measured using ImageJ 1.45s (USA).

### 2.5. Statistical Analysis

Data were presented as means ± standard deviations. Statistical analyses were performed using SPSS version 20.0 (SPSS Inc., Chicago, IL, USA). The CCS rates were compared by calculating the odds ratio (OR) and corresponding 95% confidence intervals (CI) using the crosstabs function of SPSS, which permits the assessment of significant differences between the two parameters using chi-square test. The significance of the differences between the two groups was determined with *t*-test. A *P* value of <0.05 was considered statistically significant in all cases.

## 3. Results

### 3.1. Clinical Characteristics of Patients with Capsular Contraction Syndrome

**Figure 1:**
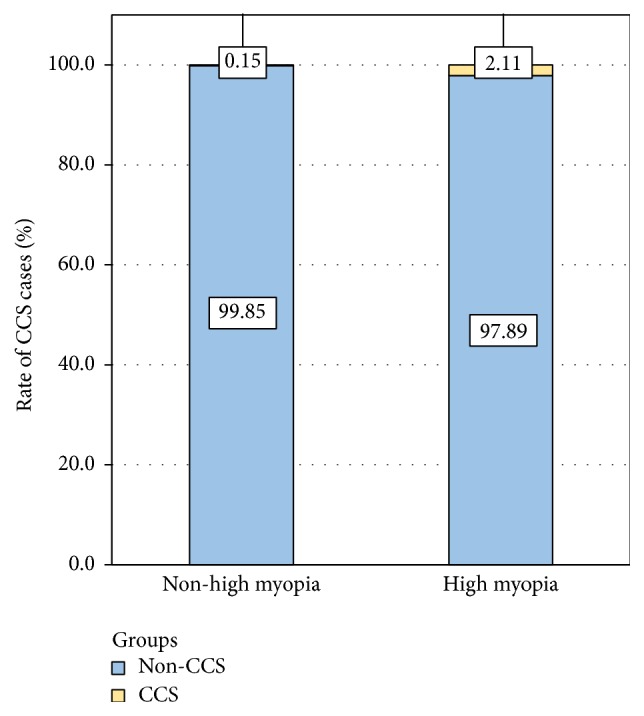
Incidence of capsular contraction syndrome in cataract patients with or without high myopia. CCS = capsular contraction syndrome.

Between September 1, 2012, and March 31, 2013, 4096 patients underwent cataract surgery at our hospital, including 665 patients with and 3431 without high myopia (Table 1). All the CCS cases were diagnosed within 3 months after cataract surgery, with 14 HMC and 5 ARC (Table 2). Our study revealed that patients with HMC were at significantly higher risk of developing CCS (Figure 1) compared with non-HMC patients, with a summary odds ratio of 14.74 (*P* < 0.001; 95% CI: 5.29–41.05; chi-square test). No significant differences were seen between patients' age and gender. Compared with the ARC group, the interval between cataract surgery and the onset of CCS was relatively shorter in HMC groups, however, without statistical significance (both *P* > 0.05, *t*-test).

### 3.2. Histological Staining of Anterior Capsular Membranes of CCS Patients

**Figure 2:**
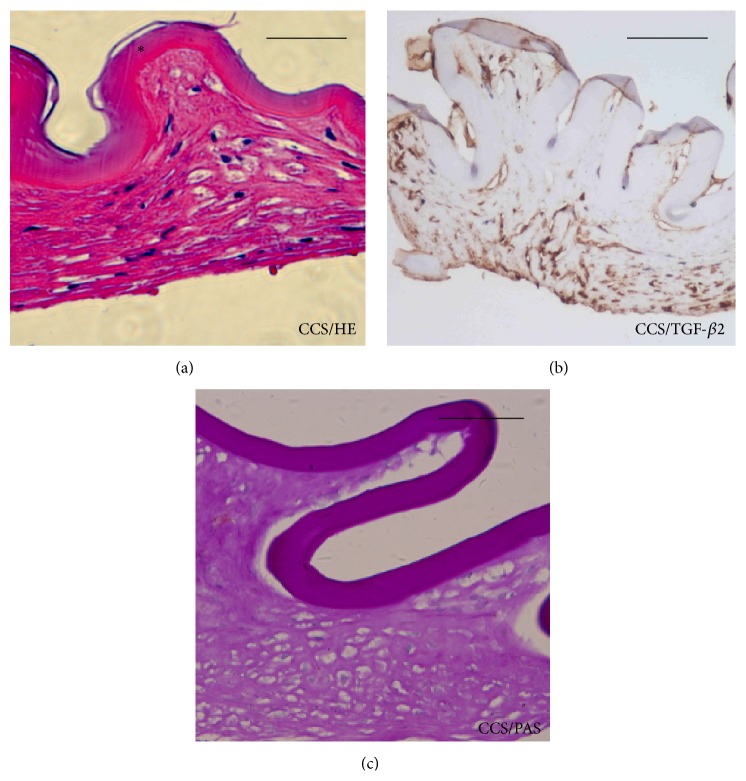
Histopathological analysis of the lens anterior capsular membrane in high myopic capsular contraction syndrome (CCS). HE and PAS staining revealed thickening of the anterior capsular membrane with spindle-like cells in high myopic CCS cases. Immunohistochemical staining of transforming growth factor-*β*2 (TGF-*β*2) in CCS cases showed diffuse intense signals located beneath the anterior capsular membrane (asterisk). Scale bar = 100 *μ*m. CCS = capsular contraction syndrome.

HE staining revealed thickening of membranous tissue and altered cellular morphology in the eyes of all CCS cases (Figure 2). The shrunken membrane consisted of multiple layers of spindle-like LECs proliferating on the inner surface, with a tendency for endocentric outgrowth from the margin and excessive fibrous extracellular matrix. These multilayered regions also showed strong TGF-*β*2 immunoreactivity (Figure 2).

### 3.3. TGF-*β*2 Concentrations in the Aqueous Humor

**Figure 3:**
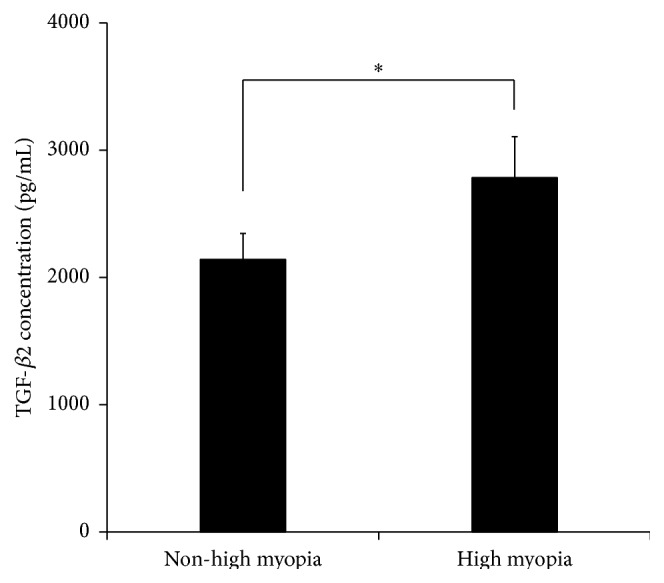
TGF-*β*2 concentration in aqueous humor of capsular contraction syndrome. ELISA results showed significantly higher levels of average TGF-*β*2 in the aqueous humor of CCS patients with high myopia than in CCS patients with ARC (*P* < 0.001, *t*-test). ELISA = enzyme-linked immunosorbent assay; TGF-*β*2 = transforming growth factor-*β*2; CCS = capsular contraction syndrome.

**Figure 4:**
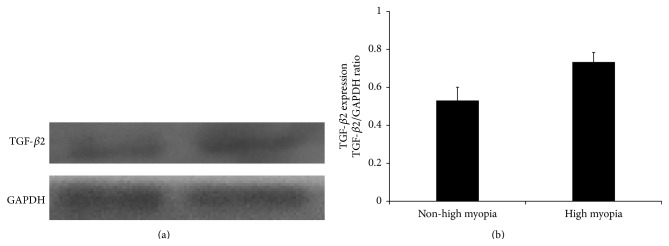
TGF-*β*2 expression in the aqueous humor of capsular contraction syndrome patients. Western blot of CCS cases showed higher TGF-*β*2 levels in the CCS with high myopia compared with those without high myopia, consistent with our previous ELISA results. TGF-*β*2 = transforming growth factor-*β*2; CCS = capsular contraction syndrome.

ELISA analysis showed that the average concentration of TGF-*β*2 in the aqueous humor of CCS patients with high myopia was 2784 ± 322 pg/mL, which was significantly higher than in CCS patients with ARC (*P* < 0.001, *t*-test; Table 2 and Figure 3).  Figure 4 shows the western blot results of TGF-*β*2 in aqueous humor. TGF-*β*2 expression varied between CCS patients with ARC and HMC, consistent with ELISA results.

## 4. Discussion

Capsular contraction syndrome (CCS) is an uncommon complication of cataract surgery. The onset of CCS in previously reported cases ranged from weeks to years after uneventful cataract surgery [13], with adverse events including blurry vision, an eccentric IOL, and full flexion of the haptics [3].

Previous studies reported a few representative cases of CCS after capsulorhexis and its treatment in patients with pseudoexfoliation [1] and retinitis pigmentosa [11]. However, the CCS cases in cataract patients with high myopia were seldom reported. Although myopia is the most common worldwide ocular disorder, the prevalence of myopia is much higher in Asians than in Caucasians [14]. The geographical differences between Asian and Western countries may be the underlying factors contributing to the differences in composition of the populations affected.

Therefore, we investigated the possible correlation between CCS and high myopia, in the absence of studies reporting this issue. Our data suggest that the prevalence of CCS was higher in patients with HMC than in ARC. Further statistical analysis showed that the patients with HMC were at a significantly higher risk of developing CCS, compared with non-HMC patients, with a summary OR of 14.74 (*P* < 0.001, 95% CI: 5.29–41.05). Therefore, our study reveals that high myopia is a risk factor for CCS. It is the first study of prevalence rate and risk analysis of CCS in HMC patients, based on a large sample of over 4000 cataract patients. The general size of capsulorhexis size is around 5–5.5 mm adjusted to the diameter of the optic surface of intraocular lens [13]. However, these HMC patients may show a higher possibility of developing exaggerated reduction in anterior capsulorhexis and capsule contraction. In order to reduce the incidence of CCS after capsulorhexis in HMC patients at higher risk, we recommend considering a relatively larger size (5.5–6 mm) of continuous curvilinear capsulorhexis during cataract surgery. Further studies are needed to determine the appropriate capsulorhexis size in HMC patients to reduce the rate of CCS after cataract surgery.

Further, both ELISA and western blot of CCS cases showed that TGF-*β*2 concentrations in CCS with high myopia were higher than those without high myopia. Elevated TGF-*β*2 may be considered as an important factor contributing to the pathogenesis of CCS. TGF-*β*2 is a central mediator of fibrosis [10], which is considered the most important predisposing factor for excessive capsular contraction, owing to its role in cellular differentiation, inflammation, and tissue repair [15, 16]. Yata et al. [17] confirmed the antifibrotic effect of TGF-*β*2 inhibition in chronic hepatic injury. Wormstone [8] demonstrated* in vitro* that the addition of TGF-*β*2 accelerated lens epithelial-myofibroblast transdifferentiation and contraction of the capsular bag.

In our present study, we also discussed the etiology of CCS in 5 patients without high myopia based on clinical and experimental data. Previous studies reported that the pathogenesis of capsular contraction syndrome involved anterior lens epithelial cells (LECs) myofibroblastic metaplasia and contraction of the fibrous membrane, as well as its outgrowth from the capsule margin [1, 2]. It was strongly correlated with the surgical technique of posterior membrane polishing during phacoemulsification and IOL implantation. Several conditions (pseudoexfoliation syndrome, uveitis, advanced age, retinitis pigmentosa, trauma, and diabetes mellitus) leading to instability of the blood-aqueous barrier have been identified as risk factors for the development of capsular contraction syndrome [3]. However, the five ARC cases were not associated with the above diseases or trauma except for age differences. Our statistical analysis revealed no significant differences in age between the two CCS groups.

There are also other aspects to this study that need to be interpreted with some caution. Firstly, we failed to compare IOL design and materials in our study, which may need to be improved. A few studies suggested that the IOL design and material were also correlated with capsular contraction, including cases of acrylic-preloaded IOL implantation. Secondly, the numbers of CCS patients is relatively small at present in our study. However, based on this preliminary study on the rate of CCS after cataract surgery in our medical centre, we were able to obtain those fundamental and indispensable data to follow-up and investigate more CCS patients after cataract surgery in our future study. Thirdly, we failed to consider the posterior capsule opacification (PCO) stage in our study. According to a recent animal model, progression of anterior capsule contraction and PCO was less likely in aphakic eyes than in IOL-implanted eyes [18]. Another study investigated the development of capsular bag opacification in rabbit eyes after implantation of an IOL designed to minimize contact between the anterior capsule and the IOL and ensure expansion of the capsular bag and found that anterior capsular contraction (ACO) and PCO were both detected during follow-up [19]. Indeed, PCO and ACO shared a common fibrosis process. Residual lens epithelial cells displayed a wound-healing response following surgery, which eventually resulted in a secondary loss of vision characterized by fibrosis, including hyperproliferation, migration, matrix deposition, matrix contraction, and transdifferentiation into myofibroblasts [20], resulting in capsular contraction and PCO.

We suggested that increased levels of TGF-*β*2 in the aqueous humor of high myopic eyes may be attributed to the unique intraocular microenvironment, since high TGF-*β*2 expression in other regions of high myopic eyes, such as sclera [21] and retina-retinal pigment epithelium-choroid [22], was also reported previously. It is possible that the unique microenvironment associated with elevation of TGF-*β*2 in high myopic eyes may explain the consequently increased risk of CCS.

In conclusion, TGF-*β*2 plays an important role in the pathogenesis of CCS, which is associated with a higher risk of high myopia.

## Figures and Tables

**Table 1:** Number of CCS cases among cataract patients with or without high myopia at the Eye and ENT Hospital, Fudan University, Shanghai, China, between September 1, 2012, and March 31, 2013.

Patients	Non-CCS	CCS	Total
Non-high myopia	3426	5	3431
High myopia	651	14	665

Total	4077	19	4096

CCS = capsular contraction syndrome.

**Table 2:** Clinical profile of CCS patients.

	CCS patients	
	ARC	HMC
Number of eyes	5	14
Age (y)	60.8 ± 6.3	59.9 ± 5.6
Gender (male/female)	2/3	6/8
Axial length (mm)	23.6 ± 0.7	31.5 ± 2.1
Interval time (days)^*∗*^	67.6 ± 17.8	50.8 ± 9.1
TGF-*β*2 (pg/mL)	2141 ± 205	2784 ± 322

CCS = capsular contraction syndrome; ARC = age-related cataract; HMC = high myopic cataract; TGF-*β*2 = transforming growth factor-*β*2.

^*∗*^Interval between cataract surgery and the onset of capsular contraction syndrome.
